# The aryl hydrocarbon receptor regulates nucleolar activity and protein synthesis in MYC-expressing cells

**DOI:** 10.1101/gad.313007.118

**Published:** 2018-10-01

**Authors:** M. Carmen Lafita-Navarro, Min Kim, Nofit Borenstein-Auerbach, Niranjan Venkateswaran, Yi-Heng Hao, Roshni Ray, Thomas Brabletz, Pier Paolo Scaglioni, Jerry W. Shay, Maralice Conacci-Sorrell

**Affiliations:** 1 Department of Cell Biology, University of Texas Southwestern Medical Center, Dallas, Texas 75390, USA;; 2 Lyda Hill Department of Bioinformatics, University of Texas Southwestern Medical Center, Dallas, Texas 75390, USA;; 3 Nikolaus-Fiebiger-Center for Molecular Medicine, University Erlangen-Nurnberg, 91054 Erlangen, Germany;; 4 Department of Medicine, Division of Hematology Oncology, University of Cincinnati College of Medicine, Cincinnati, Ohio 45267, USA;; 5 Harold C. Simmons Comprehensive Cancer Center, University of Texas Southwestern Medical Center, Dallas, Texas 75390, USA

**Keywords:** AHR, Myc, aryl hydrocarbon receptor, cancer, nucleolus, ribosome biogenesis, transcription, translation

## Abstract

MYC enhances protein synthesis by regulating genes involved in ribosome biogenesis and protein translation. Here, we show that MYC-induced protein translation is mediated by the transcription factor aryl hydrocarbon receptor (AHR), which is induced by MYC in colonic cells. AHR promotes protein synthesis by activating the transcription of genes required for ribosome biogenesis and protein translation, including OGFOD1 and NOLC1. Using surface sensing of translation (SUnSET) to measure global protein translation, we found that silencing AHR or its targets diminishes protein synthesis. Therefore, targeting AHR or its downstream pathways could provide a novel approach to limit biomass production in MYC-driven tumors.

## Abstract (precis)

Lafita-Navarro et al. show that MYC-induced protein translation is mediated by the transcription factor aryl hydrocarbon receptor (AHR), which is induced by MYC in colonic cells.

Increased ribosome biogenesis and protein translation are essential for cell growth and proliferation. Because ribosomal RNA (rRNA) transcription, rRNA processing, and ribosome subunit assembly occur in the nucleolus, nucleolar size, number, and activity are often increased in cancer cells (Pelletier et al. 2018). Numerous reports have shown that deregulated MYC leads to an increase in the expression of genes involved in protein synthesis and ribosome biogenesis (van Riggelen et al. 2010). MYC was shown to promote ribosome biogenesis by directly binding to the rDNA promoter and enhancing rRNA transcription, by accelerating rRNA processing, and by augmenting polymerase III (Pol III)-mediated 5S and transfer RNA (tRNA) transcription (Gomez-Roman et al. 2003; Grandori et al. 2005).

MYC is the most frequently deregulated gene in human tumors and is required for initiation and maintenance of nearly all cancers (Beroukhim et al. 2010). MYC activates the expression of thousands of genes through at least three known mechanisms: (1) In complex with MAX, MYC directly induces the expression of genes that contain E-boxes in their regulatory regions. (2) MYC prevents pausing of RNA Pol II and enhances the expression of active genes (Rahl et al. 2010). (3) MYC indirectly regulates genes by controlling their transcriptional regulators (Sabo et al. 2014). Therefore, dissecting transcriptional networks downstream from MYC may lead to the identification of specific oncogenic signatures and reveal unexplored opportunities for therapeutic interventions.

The basic helix–loop–helix (bHLH)-PAS protein aryl hydrocarbon receptor (AHR) is a broadly expressed transcription factor that senses environmental toxins such as dioxins. AHR is highly expressed in the liver and lungs, where it plays a critical role in detoxifying environmental pollutants, including aromatic hydrocarbons such as benzopyrene (Tsay et al. 2013). These pollutants directly bind AHR in the cytoplasm, leading to its nuclear translocation (Knutson and Poland 1982) and heterodimerization with the AHR nuclear translocator (ARNT). In response to xenobiotics (such as TCDD), AHR:ARNT dimers induce the expression of detoxifying enzymes such as CYP1A1 and NQO1.

While most studies indicate that AHR functions as an oncogene, other studies propose that AHR can act as a tumor suppressor. For example, ablation of AHR accelerates intestinal tumor formation by stabilizing β-catenin by mechanisms that do not involve AHR's transcriptional activity (Kawajiri et al. 2009; Ikuta et al. 2013). However, AHR is not lost or repressed in tumors, thus indicating that AHR is not a canonical tumor suppressor. On the contrary, AHR was found to be highly expressed in tumors of different origins such as lung, breast, liver, ovarian, prostate, and colon (Koliopanos et al. 2002; Opitz et al. 2011). Studies of human colon cancer cells showed that AHR activation by TCDD drives colon cancer cell survival and migration (Xie et al. 2012) and that overexpression of a constitutively active AHR causes stomach cancer (Andersson et al. 2002). The role of AHR in cancer is likely to be regulated by additional factors such as its binding to specific ligands and the landscape of oncogenes/tumor suppressors of each tumor. To date, the upstream regulators of AHR in tumor cells and the specific molecular contexts in which AHR functions as an oncogene have not been identified.

## Results and Discussion

### AHR is transcriptionally induced by MYC

**Figure 1.**
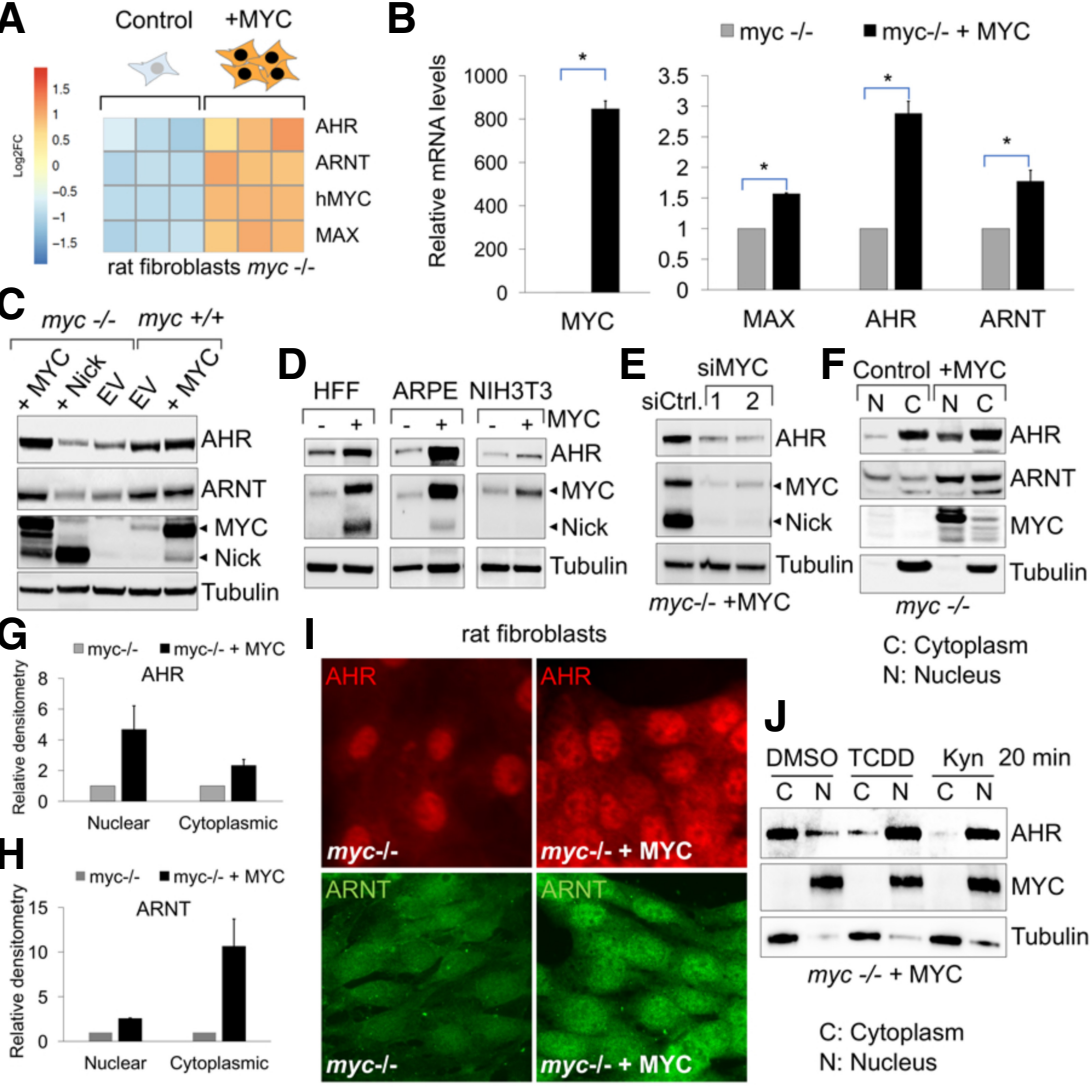
AHR expression is induced by MYC. (*A*) Heat map as determined by RNA-seq. (*B*) RT-qPCR of *myc*^−/−^ fibroblast expressing vector or MYC. (*C*) Western blot of *myc*^−/−^ fibroblasts expressing vector, MYC, or MYC-nick and wild-type fibroblasts (*myc*^+/+^) expressing empty vector or MYC. (*D*) Western blot of HFF, ARPE-I90, and NIH3T3 cells expressing MYC. (*E*) Western blot of AHR in *myc*^−/−^ cells expressing MYC transfected with siRNAs that target MYC. (*F*) Western blot of nuclear (N) and cytoplasmic (C) fractions of *myc*^−/−^ cells expressing empty vector or MYC harvested 3 d after seeding. (*G*,*H*) Quantification of nuclear and cytoplasmic pools of AHR and ARNT. *n* = 3. (*I*) Immunofluorescence for AHR and ARNT. (*J*) Western blot of nuclear (N) and cytoplasmic (C) fractions of *myc*^−/−^ cells expressing MYC after treatment with 1 nM TCDD and 20 µM kynurenine for 20 min (see Supplemental Figure S1F for quantification). (*) *P*-value < 0.05.

In an RNA sequencing (RNA-seq) experiment comparing *myc*^−/−^ (HO15.19) rat fibroblasts expressing vector or reconstituted with human MYC, we found that *AHR* and its heterodimeric partner, *ARNT*, were induced by MYC (Fig. 1A–C; Supplemental Fig. S1C–E). Gene ontology analysis of our RNA-seq results (Supplemental Fig. S1A–C) corroborated previous reports on expression changes driven by MYC, indicating that targets identified in our experiment are indeed representative of a MYC signature (Sabo et al. 2014). The up-regulation of *AHR* and *ARNT* in MYC-expressing cells was validated by using RT-qPCR (Fig. 1B) and Western blotting of *myc*^−/−^ or wild-type (*myc*^+/+^) fibroblasts overexpressing MYC (Fig. 1C) and *myc*^−/−^ cells expressing a doxycycline-inducible MYC (Supplemental Fig. S1E). While AHR expression was stimulated by full-length MYC, cells expressing the transcriptionally inactive MYC-nick exhibited no changes in their AHR levels (Fig. 1C). Additional cell lines, including the human retinal pigment epithelial line ARPE-I90 and the fibroblasts HFF and NIH3T3, also displayed increases in AHR upon MYC overexpression (Fig. 1D). Conversely, silencing MYC by siRNA reduced AHR (Fig. 1E).

AHR levels were elevated in the nuclei of MYC-expressing cells (Fig. 1F,G,I), indicating that AHR is transcriptionally active in these cells. Nuclear ARNT levels were also elevated by MYC but to a lesser extent than AHR (Fig. 1F,H,I). The addition of AHR ligands, such as the tryptophan metabolite kynurenine (Kyn) and TCDD, to the culture medium further increased its nuclear translocation (Fig. 1J; Supplemental Fig. S1F).

### AHR stimulates the transcription of a subset of genes in MYC-expressing cells

**Figure 2.**
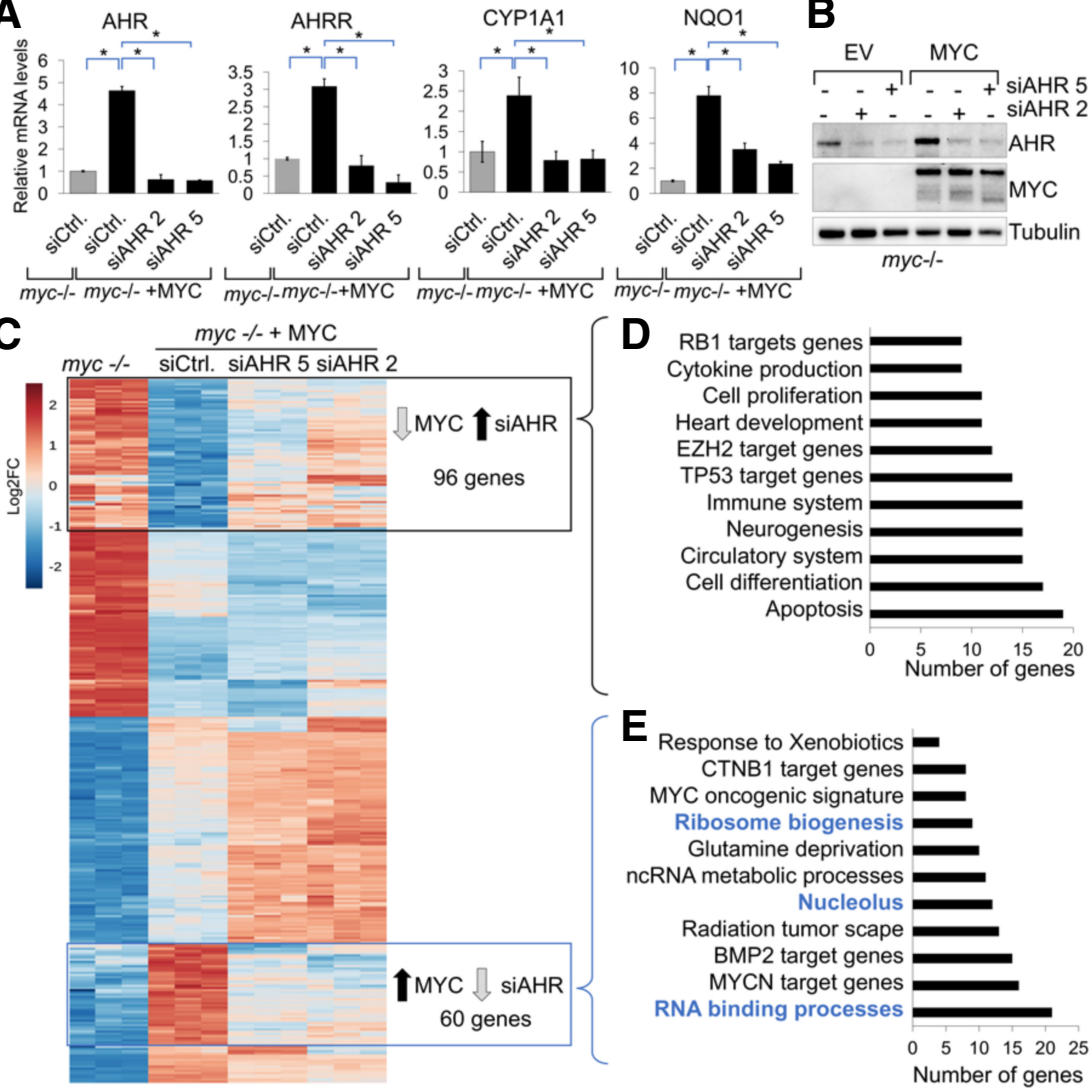
A subset of MYC-regulated genes is dependent on AHR. (*A*) RT-qPCR for AHR and the AHR target genes in *myc*^−/−^ cells or *myc*^−/−^ + MYC 48 h after AHR siRNA transfection. (*) *P*-value < 0.05. (*B*) Western blot showing AHR silencing in *myc*^−/−^ cells expressing vector or MYC 48 h after transfection. (*C*) Heat map comparing the transcriptional signature of *myc*^−/−^ cells expressing vector, MYC, and MYC transfected with two siRNAs for AHR. (*D*) Gene ontology of MYC-repressed genes derepressed by AHR knockdown. (*E*) Gene ontology of MYC-activated genes that were repressed by AHR knockdown (also see Supplemental Fig. S3A,B).

In agreement with an increase in the nuclear pool of AHR, we found that canonical AHR target genes such as *AHRR*, *CYP1A1*, and *NQO1* were up-regulated in MYC-expressing cells (Fig. 2A). Furthermore, this up-regulation was significantly attenuated by knocking down *AHR* (Fig. 2A). Importantly, AHR knockdown did not affect MYC levels (Fig. 2B). To determine the extent to which AHR stimulates the transcription of genes in MYC-expressing cells, we performed RNA-seq comparing *myc*^−/−^ cells expressing a vector control or MYC 48 h after AHR knockdown, when silencing was effective (Fig. 2B) but cell survival was not impaired (Supplemental Fig. S2A). The technical reliability of our RNA-seq experiment was first confirmed by examining the expression of well-known MYC-induced genes (*CAD*, *NPM1*, *RCC*, *LDHA*, *CCNA2*, *NCL*, and *CDC25a*) and a MYC-repressed gene (*SRD5A1*) (Supplemental Fig. S2B). As expected, the expression of canonical AHR targets (*AHRR*, *ARNT*, *CYP1A1*, and *NQO1*) in MYC-expressing cells was dependent on AHR (Supplemental Fig. S2B).

To quantify the contribution of AHR to MYC-regulated genes, we first selected genes that were differentially regulated by MYC. Applying a cutoff of log_2_ fold change of ≥0.7 or −0.7 or less and an adjusted *P*-value of ≤0.05, we found that 4970 genes were differentially regulated by MYC, with roughly equal numbers of genes being up-regulated and down-regulated. Among the MYC-regulated genes, 292 genes were affected by at least 1.3-fold upon AHR silencing with two independent siRNAs using an adjusted *P*-value of ≤0.05 (Fig. 2C). A portion of AHR-regulated genes overlapped with genes previously shown to be driven by MYC (Supplemental Fig. S2C; Sabo et al. 2014). Gene ontology analysis of targets repressed by MYC, which were derepressed by AHR silencing, identified signatures of RB1 and P53 targets as well as apoptosis and lineage-specific genes (Fig. 2D; Supplemental S2D,E, Supplemental S3A). The induction of these genes by AHR knockdown was expected because the reduction of AHR levels has been shown to promote apoptosis and differentiation (Apetoh et al. 2010; Zhu et al. 2012; Hughes et al. 2014).

### AHR regulates the expression of genes involved in protein synthesis

**Figure 3.**
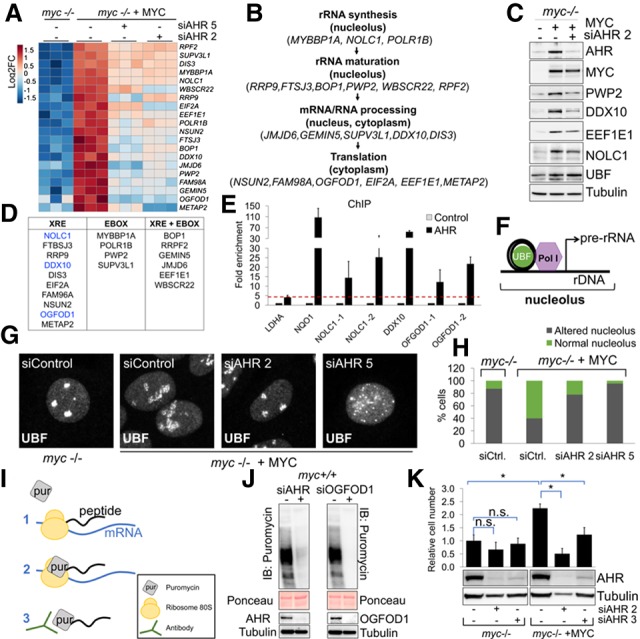
AHR regulates genes involved in protein synthesis. (*A*) Heat map of genes involved in translation induced by MYC and repressed by siAHR found through RNA-seq. (*B*) AHR-regulated genes involved in protein synthesis (also see Supplemental Fig. S4B). (*C*) Western blot of genes shown in *A* and upstream binding factor (UBF) in *myc*^−/−^ cells expressing empty vector or MYC 72 h after transfection of siRNA. (*D*) Table showing the presence of canonical E-box or XRE in the promoters of the genes found in *A* (also see Supplemental Fig. S5). (*E*) ChIP of AHR on the promoters of genes shown in *A* (also see Supplemental Fig. S6A). The red line defines the positive immunoprecipitated signal. (*F*) UBF and RNA Pol I on the rDNA promoter. (*G*) Immunofluorescence for UBF 72 h after transfection with control or AHR siRNAs. (*H*) Quantification of *G* (also see Supplemental Fig. S6B). (*I*) Surface sensing of translation (SUnSET) method to measure protein translation rate. (*J*) Forty-eight hours after transfection with siRNA for AHR or OGFOD1, cells were serum-starved overnight, and FBS and puromycin were added for 1 h prior to harvesting for puromycin Western blotting. (*K*) Relative proliferation of *myc*^−/−^ rat fibroblasts expressing vector or MYC transfected with control siRNA or AHR siRNA and counted 6 d after transfection (also see Supplemental Fig. S4C).

To identify genes directly induced by AHR in MYC-expressing cells, we analyzed genes that were up-regulated by MYC and repressed upon *AHR* silencing. This signature of AHR-regulated genes included previously known *MYC* and *MYCN* target genes (i.e.: *NOLC1*, *POLR1B*, or *NDRG1*), implicating AHR as mediator of MYC functions. Our analysis revealed that genes responsible for ribosome biogenesis, nucleolar function and composition, and RNA binding and processing were highly represented among AHR targets (Fig. 2E, Supplemental Figs. S3B, S4A,B). This was particularly interesting because MYC expression has been strongly correlated with increased ribosome biogenesis and elevated protein synthesis in cancer cells (Grandori et al. 2005). We validated the RNA-seq results by Western blotting for periodic tryptophan protein (PWP2), a component of the small subunit of the rRNA processome; DEAD-box helicase 10 (DDX10); the eukaryotic translation elongation factor 1ε1 (EEF1E1); and the nucleolar protein NOLC1 (Fig. 3C; Supplemental Fig. S4E,F). Of the 20 AHR-regulated genes, 16 contain XRE/HRE elements on their promoters (Fig. 3D; Supplemental Fig. S5), suggesting that these are directly activated by AHR. We randomly chose three genes (Supplemental Fig. S6A) and verified by chromatin immunoprecipitation (ChIP) with an anti-AHR antibody that AHR binds to their XRE elements. We used the canonical AHR target gene NQO1 as a positive control (Fig. 3E).

Because our RNA-seq data indicated that AHR induced the expression of genes involved in ribosome biogenesis (Fig. 3B), we examined nucleolar morphology and transcriptional activity by performing immunofluorescence for the nucleolar transcription factor UBF (upstream binding factor) (Fig. 3G). UBF expression was not affected by AHR (Fig. 3C). MYC-expressing cells display large and open nucleolus morphology, an indication of highly active ribosome biogenesis (Boulon et al. 2010). Upon *AHR* silencing, nucleolar morphology resembled inactive or disassembled nucleoli (Fig. 3G,H; Supplemental Fig. S6B). These results suggest that AHR is involved in nucleolar activity and protein synthesis in MYC-expressing cells.

To determine whether AHR regulates protein translation in MYC-expressing cells, we used a nonradioactive method named SUnSET (surface sensing of translation) that allows monitoring and quantification of global protein synthesis (Fig. 3I; Schmidt et al. 2009). We performed SUnSET on cell lines that did not undergo significant cell death upon AHR silencing (Supplemental Fig. S4D) and found that AHR knockdown reduced protein synthesis (Fig. 3J). Similarly, silencing AHR target genes such as OGFOD1 also reduced protein synthesis (Fig. 3J), which is in agreement with previous studies showing that OGFOD1 is involved in mRNA translation (Singleton et al. 2014). Using Coomassie staining of a gel loaded with protein lysates corresponding to equal cell numbers, we found that MYC promoted an increase in total protein levels, and silencing AHR dampens this increase (Supplemental Fig. S4G,H). Silencing AHR caused the death of MYC-expressing cells but not of *myc*^−/−^ cells (Fig. 3K). AHR knockdown also reduced the viability of wild-type fibroblasts overexpressing MYC, thus confirming that ectopic expression of MYC sensitizes rat fibroblast cells to AHR depletion (Supplemental Fig. S4D). Our results indicate that AHR likely promotes biomass production in MYC-dependent cells by increasing protein translation.

### MYC drives the expression of AHR in colon cancer cells

**Figure 4.**
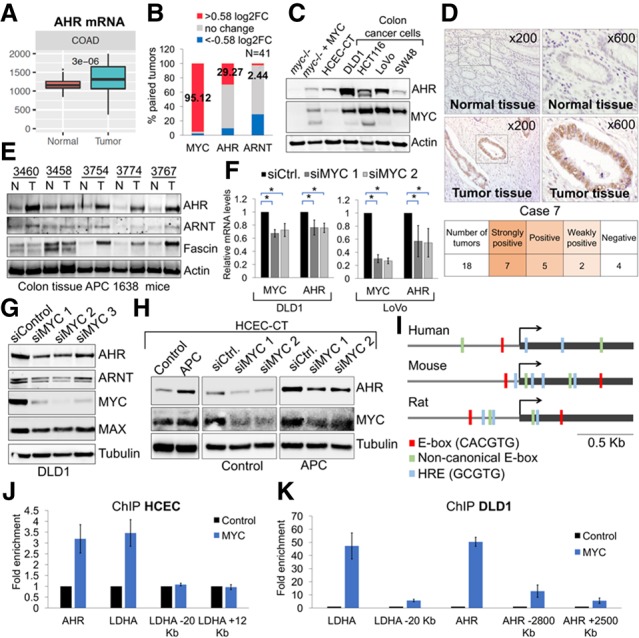
AHR is induced by MYC in colon cancer cells. (*A*) AHR mRNA expression comparing normal and tumor patients in TCGA. *n* = 41 normal samples; *n* = 478 tumor samples. (*B*) Log_2_ fold change (log2FC) in MYC, AHR, and ARNT mRNA levels of tumor samples normalized to patient-matched benign tissue. (*C*) Western blot of colon cancer cells lines and HCECs (see Supplemental Fig. S7A,B for quantification). (*D*) Immunohistochemistry analysis of AHR in patient-matched colon cancer tissue. *n* = 18. (*E*) Western blot of tumor and neighboring normal mucosa derived from the CPC APC mouse model of colon cancer. (*F*,*G*) RT-qPCR and Western blot for MYC, AHR, and ARNT in colon cancer cells transfected with MYC siRNAs (see Supplemental Fig. S8C for quantification). (*H*) Western blot of control (CT) or APC-expressing (CTA) HCECs transfected with control and MYC siRNAs 72 h after transfection (also see Supplemental Fig. S8D–F for quantification). (*I*) Representation of 1 kb upstream of or downstream from the transcription start site (TSS) of AHR. (*J*) ChIP for MYC (N-262 antibody, Santa Cruz Biotechnology) on the promoter regions of AHR and LDHA or on control regions lacking E-boxes (−20-kb or +12-kb TSS LDHA) in HCECs. LDHA was used as positive control for MYC immunoprecipitation. (*K*) ChIP for MYC (Y69 antibody, Abcam) on the promoter regions of AHR and LDHA and two surrounding upstream and downstream regions that do not contain E-boxes (−2800-kb or +2500-kb TSS AHR and −20-kb TSS LDHA). Fold enrichment for MYC immunoprecipitation in comparison with control (IgG in *J* or no antibody in *K*) was determined by qPCR.

Given that MYC is required for the initiation and progression of multiple types of cancers and that increased protein translation is necessary for the process of oncogenic transformation, we asked whether AHR levels are greater in human tumors than normal tissues. By analyzing The Cancer Genome Atlas (TCGA) database, we found that *AHR* mRNA was elevated in colorectal adenocarcinoma (COAD) (Fig. 4A). Comparing mRNA levels for *MYC* and *AHR* in normal and tumor pairs of samples present in TCGA (*n* = 41), we found that >95% of the colon cancer tumors displayed >1.5-fold change in MYC mRNA levels (Fig. 4B), and 29.3% of the normal tumor pairs exhibited an increase of >1.5-fold-change in AHR mRNA (Fig. 4B). Nearly half (48.78%) of colon cancer patients had at least a 1.3-fold increase in AHR levels (data not shown). In agreement with TCGA data, AHR protein levels were elevated in the majority of colon cancer cell lines analyzed, when compared with normal human colonic epithelial cells (HCECs) that were immortalized by hTERT and CDK4 (Fig. 4C; Roig et al. 2010).

Immunohistochemistry (IHC) on 18 paraffin-embedded normal and colon cancer tissue samples from patients demonstrated that AHR expression was significantly higher in 14 out of 18 samples (Fig. 4D; Supplemental Fig. S8). Because MYC is necessary for initiation of colon cancer in a mouse model driven by the Wnt pathway (Sansom et al. 2007), we determined the levels of AHR in mice expressing an oncogenic truncated APC variant that activates the Wnt pathway. These animals develop colorectal carcinoma that mimics human disease (Hinoi et al. 2007). Consistent with our finding, AHR expression was greater in tumor tissues than in the healthy tissues (Fig. 4E). Knocking down MYC down-regulated AHR mRNA and protein in colon cancer cells (Fig. 4F,G), while overexpression of MYC induced AHR (Supplemental Fig. S9A). MYC stabilization due to the knockout of its E3 ligase FBW7 was also correlated with increased AHR in colon cancer cells (Supplemental Fig. S9B).

Expressing an oncogenic truncated variant of APC in HCECs also led to the up-regulation of AHR (Fig. 4H). Silencing MYC caused a down-regulation of AHR in normal HCECs and HCECs transformed by truncated APC (Fig. 4H). This is in agreement with our results in the colon tissues of animals expressing truncated APC (Fig. 4E). Our results indicate that MYC is a global activator of AHR in normal and cancer cells and that oncogenic signals that increase MYC levels or activity likely promote an increase in AHR.

Bioinformatics analysis identified a conserved canonical E-box sequence in the *AHR* promoter, an indicator of a MYC-binding site (Fig. 4I). Using ChIP, we demonstrated that the E-box on the *AHR* promoter was specifically bound by MYC (Fig. 4J,K; Supplemental Fig. S7H) and its heterodimeric partner, MAX (Supplemental Fig. S7G). The canonical MYC target gene *LDHA* was used as a positive control. Indeed, the ENCODE database documented binding of MYC to the human *AHR* promoter in cell lines of different origins (Supplemental Fig. S7J), thus providing further evidence that MYC is a transcriptional regulator of *AHR*. While ENCODE data indicate that MYC also binds to the *ARNT* promoter, we did not find a statistically significant increase in the binding of MYC to the *ARNT* promoter (Supplemental Fig. S7H–J).

### AHR regulates the expression of genes involved in protein synthesis in colon cancer cells

**Figure 5.**
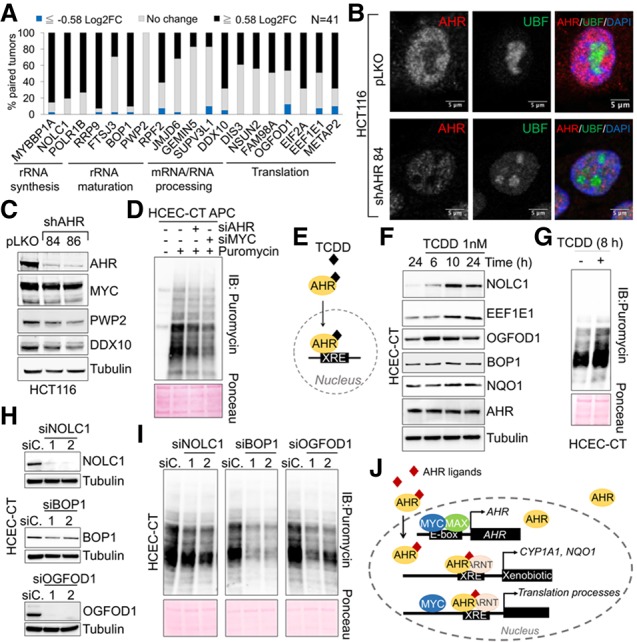
AHR target genes involved in protein synthesis are induced in human colon cancer tissues. (*A*) The percentage of tumors with different log_2_ fold change (Log2FC) ratios in mRNA levels for AHR-regulated genes. Data were obtained from TCGA. *n* = 41 pairs. (*B*) Immunofluorescence for UBF on HCT116 cells infected with control (pLKO) or shAHR (see Supplemental Fig. S9D for quantification). (*C*) Western blot of HCT116 control (pLKO) or shAHR. (*D*) Western blot for puromycin of APC HCECs after SUnSET. Cells were transfected with siRNA for AHR or MYC. Forty-eight hours later, cells were serum-starved overnight, and then FBS and puromycin were added for 1 h prior to harvesting for Western blot. (*E*) AHR nuclear translocation and activation upon exposure to the oncogenic ligand TCDD. (*F*) Western blot of control (CT) HCECs after treatment with 1 nM TCDD for 6, 10, and 24 h or with DMSO for 24 h. (*G*) Western blot for puromycin of control (CT) HCECs after SUnSET in cells treated with 1 nM TCDD for 8 h. Puromycin was added for 1 h, and cells were harvested for SUnSET. (*H*) Western blot for NOLC1, BOP1, or OGFOD1 silencing 3 d after transfection. (*I*) Western blot for puromycin of control (CT) HCECs after SUnSET in NOLC1, BOP1, and OGFOD1 silenced cells (shown in *H*). The experiment was performed as in *D*. (*J*) Model for the cross-talk between AHR and MYC.

To examine the physiological relevance of AHR-regulated genes in colon cancer cells, we probed the TCGA database for the expression of the AHR-dependent target genes induced in MYC-expressing cells. AHR-regulated genes involved in rRNA production and protein translation are up-regulated in tumor samples when compared with normal tissues from the same patients (Fig. 5A). As expected, the AHR-regulated gene signature was highly increased in AHR-positive tumor samples (Supplemental Fig. S9F). AHR knockdown in colon cancer cells prevented the accumulation of UBF in the nucleolus (Fig. 5B; Supplemental Fig. S9D) and reduced the levels of AHR targets PWP2 and DDX10 (Fig. 5C; Supplemental Fig. S9B). Moreover, AHR silencing in APC transformed HCECs reduced protein translation (Fig. 5D), thus confirming that protein translation is regulated by AHR.

Activation of AHR with TCDD (Fig. 5E) in HCECs induced the expression of AHR target genes such as NOLC1, EEF1E1, and OGFOD1 (Fig. 5F). In addition, TCDD treatment was capable of enhancing protein translation (Fig. 5G). Silencing the TCDD-activated AHR target genes was sufficient to reduce protein synthesis (Fig. 5H,I). While silencing AHR had no significant effect on the viability of colon cancer cells (data not shown), it invariably caused an increase in p27 levels (Supplemental Fig. S9E), thus indicating that colon cancer cells activate pathways that prevent cell cycle progression and proliferation upon AHR knockdown.

In summary, our results suggest that by up-regulating the expression of AHR, MYC increases the production of ribosomes and proteins to facilitate cell proliferation (Fig. 5J). Future experiments targeted at elucidating the in vivo contribution of AHR, ARNT, AHR ligands, and AHR target genes to MYC-driven tumorigenesis will be critical and may lead to novel approaches to target MYC-dependent tumors.

## Materials and methods

Cells were cultured in DMEM with 10% FBS and 100 U/mL penicillin/streptomycin. Stable cell lines were generated via retroviral or lentiviral infection. For knockdowns, 5 × 10^4^ cells were seeded in six-well plates and, the next day, transfected with 40 nM siRNA. siRNA sequences, antibodies, and primers are described in Supplemental Tables S1–S4. Cell proliferation was measured by plating 50,000 cells, transfecting them with siRNA, and directly counting them or staining them with crystal violet. Quantification was performed by diluting the incorporated crystal violet dye with 10% acetic acid and measuring absorbance at 595 nm. Protein synthesis was measured using SUnSET (Schmidt et al. 2009). This method is based on the ability of puromycin to incorporate into nascent peptides (Schmidt et al. 2009). Cells were treated with puromycin at 10 µg/mL for 1 h and lysed for use in Western blotting with a puromycin antibody. Nuclear and cytoplasmic fractionation (Conacci-Sorrell et al. 2010), immunofluorescence (Lafita-Navarro et al. 2016), disrupted nucleolar morphology (Gonyo et al. 2017), and IHC (Conacci-Sorrell et al. 2014) were performed as described previously. ChIP was performed with purified nuclei as described previously (Garcia-Sanz et al. 2014). RNA-seq assessment of the raw sequencing reads was done using NGS QC Toolkit (Patel and Jain 2012). The reads were aligned to the genome RGSC 6.0/rn6 using HISAT2 (version 2.1.0) aligner. A minimum read count filter of 10 total reads was applied to remove low-expressed genes. Filtered reads were then normalized using DESeq2 (Love et al. 2014). DeSeq2 uses a negative binomial distribution to estimate data variability and uses an error model for a more robust statistical test for significance. A false discovery rate cutoff of <5% was used to select significantly altered genes between experiment conditions. For TCGA analyses, significance testing between groups was performed using nonparametric Kruskal-Wallis test.

## Supplementary Material

Supplemental Material
