# Supplementary material for: The aryl hydrocarbon receptor regulates nucleolar activity and protein synthesis in MYC-expressing cells
Source: Genes Dev. 2018 Oct 1;32(19-20):1303–8. doi: 10.1101/gad.313007.118 (PMC6169836; doi:10.1101/gad.313007.118)
Supplement: Supplemental Material [file supp_32_19-20_1303__index.html]

The aryl hydrocarbon receptor regulates nucleolar activity and protein synthesis in MYC-expressing cells — Supplemental Material 

# The aryl hydrocarbon receptor regulates nucleolar activity and protein synthesis in MYC-expressing cells

## Supplemental Material

- Supplementary\_tableS8.xlsx
- Supplementary\_tableS5.xlsx
- Supplementary\_tableS7.xlsx
- Supplementary\_tableS6.xlsx
- Supplementary\_tableS4.xlsx
- Supplementary\_tableS3.xlsx
- Supplementary\_tableS2.xlsx
- Supplementary\_tableS1.xlsx
- Supplementary\_figures\_and\_legends.pdf
