# Supplementary material for: The aryl hydrocarbon receptor regulates nucleolar activity and protein synthesis in MYC-expressing cells
Source: Genes Dev. 2018 Oct 1;32(19-20):1303–8. doi: 10.1101/gad.313007.118 (PMC6169836; doi:10.1101/gad.313007.118)

SUPPLEMENTARY FIGURES LEGENDS and TABLES

Figure S1. RNA-seq comparing *myc-/-* cells and *myc-/-* +MYC. (A) RNA-seq analysis showing heatmap of genes differentially regulated in myc-/- fibroblast expressing empty vector or human MYC. Data show three biological replicates. (B) Numbers of up and downregulated genes obtained in the RNA-seq described in (A). (C) Top pathways regulated by MYC in rat fibroblasts. (D) Heatmap showing the regulation of PAS-domain containing transcription factors by MYC. (E) Western blot analysis for AHR, MYC and Tubulin in HO15.19 rat myc-/- cells expressing Dox-inducible MYC grown with 5 µg/mL of doxycycline for 48 h or in HO15.19 rat myc-/- or myc -/- + MYC cells. (F) Quantification of Fig. 1J Western blot.

Figure S2. MYC expression drives a signature of AHR target genes in rat fibroblasts. (A) Relative proliferation of the cells used for RNA-seq, *myc*-/- rat fibroblasts expressing vector or MYC transfected with control siRNA or AHR siRNA and counted 48 h after transfection. (B) Heatmap showing MYC and AHR target genes in our RNA-seq following the expected transcriptional change upon MYC expression or AHR silencing. (C) Overlap of the genes found in our RNA-seq in MYC-expressing cells upon AHR silencing and the MYC-driven genes found in Sabo et al. 2014. See Table S5 for additional information. (D) Heatmap of genes involved in apoptosis processes repressed by MYC and de-repressed by siAHR found through the RNA-seq. (E) Heatmap of genes involved in differentiation processes repressed by MYC and de-repressed by siAHR found through the RNA-seq.

Figure S3. Functional analysis of AHR-driven transcriptional changes. (A) Extended graph of Fig. 2D showing the categories found in the MSigDB analysis. (B) Extended graph of Fig. 2E

showing the categories found in MSigDB analysis. The group of genes for which expression was repressed by MYC and de-repressed by AHR silencing (A) or induced by MYC and repressed by AHR silencing were loaded in the GSEA/MSigDB platform for categorization. The top most significant entries according to FDR q-value are represented. See Table S6-7 for additional information.

Figure S4. AHR regulates genes involved in protein translation processes. (A) Heatmap showing genes involved in ribosome biogenesis and protein translation processes found to be driven by MYC. (B) Categorization of the genes involved in protein translation processes for which expression was changed by AHR silencing with two different siRNA. Extension of Fig. 3B. (C) Photographs of *myc-/-* expressing empty vector or MYC cells after 48 h of control or siAHR transfection. (D) Relative proliferation of *myc+/+* expressing empty vector or MYC rat fibroblasts after AHR silencing. Proliferation was assessed by crystal violet staining and quantification. (E) Western blot for AHR, PWP2, DDX10 and Actin of ARPE-I90 expressing vector or MYC after 72 h of control or siAHR transfection. (F) Western blot for NOLC1, MYC and Tubulin of ARPE-I90 expressing vector or MYC after 72 h of control or siAHR transfection. (G) Coomassie blue protein gel staining of ARPE-I90 cells expressing vector or MYC after 72 h or control or siAHR transfection. After 48 h of transfection, cells were starved from FBS ON. The next morning FBS was added back to the cells for 6 h. After 6 h, the cells were counted and lysed. Lysate corresponding to 200,000 cells were loaded for each condition. (H) Quantification of (G) using ImageJ. n=3, p-value < 0.05.

Figure S5. Genes for protein translation process have XRE in the promoter regions. Analysis for MYC canonical binding sites (E-box: CACGTG) or AHR binding sites (XRE: GCGTG) in the promoter region (1 Kb upstream/downstream the transcriptional start site for each gene) of the genes found in our RNA-seq (Fig. 3A) involved in protein translation processes. Extended data from Fig. 3D.

Figure S6. AHR regulates protein translation. (A) Representation of the regions analyzed in the AHR (Fig. 3E) ChIP. (C) Pictures of UBF nucleolar localization indicating how the cells were classified. Representation of normal or altered nucleoli based on the UBF localization pattern. Altered nucleoli presented either condensation of UBF in the nucleolus or UBF relocalization to the nucleoplasm as is shown by inhibiting rDNA transcription with Actinomycin D.

Figure S7. MYC regulates AHR expression in colonic cells. (A) Quantification of Fig. 4C Western blot. (B) Quantification of Fig. 4C Western blot. (C) Quantification of Fig. 4G Western blot. (D-F) Quantification of Fig. 4H Western blot. (G) ChIP for MAX (using sc C-17 antibody) in DLD1 on the promoter regions of AHR and LDHA or on control regions lacking E-boxes (-20 kb or +12 kb TSS LDHA) in DLD1. LDHA was used as positive control for MAX immunoprecipitation. (H) ChIP for MYC (using sc N-262 antibody) in DLD1 on the promoter regions of AHR and LDHA or on control regions lacking E-boxes (-20 kb or +12 kb TSS LDHA) in DLD1. LDHA was used as positive control for MAX immunoprecipitation. (I) Representation of 1kb upstream the transcription start site (TSS) of ARNT promoters with XRE, and non-canonical E-box. Canonical E-boxes were not found in that region. (J) Table summarizing the

ENCODE data regarding ChIP-seq of MYC and MAX on the AHR and ARNT promoter in human cell lines of different origins.

Figure S8. IHC for AHR in human colon colorectal adenocarcinoma. (A) IHC analysis for AHR in patient-matched colon cancer tissue versus normal tissue (n = 19). Image is shown at 100X. (B) Central tumor and invasive cells display elevated and nuclear AHR (200X). (C) IHC analysis for AHR indicating how the samples were classified as positive or negative for AHR staining. (D) Table summarizing results of AHR IHC in human colorectal samples, n=18. All 18 samples were examined by IHC using two MYC antibodies and they were all positively stained (Conacci-Sorrell et al. 2014).

Figure S9. AHR regulates genes involved in protein translation in colon cancer cells. (A) Western blot for AHR, MYC, MAX and Tubulin of DLD1 overexpressing vector or MYC. (B) Western blot for AHR, MAX, MYC and Tubulin in WT and *FBW7*-/- DLD1 and HCT116 cells cultured for 4 days. (C) Western blot for AHR, MYC, PWP2, DDX10 and Tubulin of DLD1 72 h after control or siAHR transfection. (D) Quantification of Fig. 5B. The area related to UBF signal was quantified by Image J. (E) Western blot for AHR, MYC, p27 and Cyclin A1 after 72 h of control, siAHR or siMYC transfection in colon cancer cell lines DLD1, LoVo and HCT116. (F) Number of colorectal adenocarcinomas (COAD) that had high AHR mRNA levels with increased expression of the genes labelled in the x axis. Log2FC was assessed by comparing the tumor and healthy tissue (n=41).

Table S1. siRNAs used in this study

siRNAs	ID NUMBER	SPECIE
human siAHR 2	SASI_Hs02 00140198	*Homo sapiens*
human siAHR 3	SASI_Hs02 00332182	*Homo sapiens*
human siNOLC1 1	SASI_Hs01_00116300	*Homo sapiens*
human siNOLC1 2	SASI_Hs01_00116304	*Homo sapiens*
human siBOP1 1	SASI_Hs01_00156981	*Homo sapiens*
human siBOP1 2	SASI_Hs01_00156982	*Homo sapiens*
human siOGFOD1 1	SASI_Hs01_00214569	*Homo sapiens*
human siOGFOD1 2	SASI_Hs01_00214570	*Homo sapiens*
rat siAHR #2	SASI_Rn01 00112270	*Rattus novergicus*
rat siAHR #3	SASI_Rn01 00112275	*Rattus novergicus*
rat siAHR #5	SASI_Rn01 00112273	*Rattus novergicus*
rat siOGFOD1	SASI_Rn02_00228556	*Rattus novergicus*
Universal negative control #1	SIC001	
Universal negative control #2	SIC002	

Table S2: Antibodies used in this study

PROTEIN	Catalogue number	COMPANY	APPLICATION
AHR	BML-SA210-0100	ENZO	WB; IF; ChIP
ARNT	H-172/sc-5580	Santa Cruz Biotechnology	WB; IF
MYC	Y69/ab32071	Abcam	WB; ChIP
MYC	N-262/sc-764	Santa Cruz Biotechnology	ChIP
Tubulin	T6199-200UL	Sigma	WB
Actin	13E5/#4970	Cell signaling	WB
PWP2	PA5-42781	Invitrogen	WB
DDX10	PA5-71354	Invitrogen	WB
EEF1E1	PA5-55722	Invitrogen	WB
UBF	F-9/sc-13125	Santa Cruz Biotechnology	WB; IF
Fascin	(D-10): sc-46675	Santa Cruz Biotechnology	WB
p27	D69C12 XP/#3686	Cell signaling	WB
CyclinA1	722407/MAB7046	Novus Biologicals	WB
NOLC1	11815-1-AP	Proteintech	WB
OGFOD1	NBP1-83826	Novus biologicals	WB
BOP1	NBP1-21389	Novs biologicals	WB
Puromycin	MABE343	Sigma	WB

Table S3: Primers used for RT-qPCR

**Primer**	**SPECIES**	**Sequence 5'-3'**
AHR	*H.Sapiens*	F: AGCCGGTGCAGAAAACAG<br>R: CTATGCCGCTTGGAAGGAT
MYC	*H.Sapiens*	F: CACCAGCAGCGACTCTGA<br>R: GATCCAGACTCTGACCTTTTGC
AHR	*R.norvegicus*	F: CTTCAGATGCCGGCTGAG<br>R: CCTCCCTTGGAAATTCATTG
ARNT1	*R.norvegicus*	F: CCACTGCACAGGTTACATCAA<br>R: TCATCATCTGGGAGGGAGAC
MAX	*R.norvegicus*	F: GAACGAAAACGTAGGGACCA<br>R: GATCTTGCCTTCTCCAGTGC
AHRR	*R.norvegicus*	F: CTGCTTCATTTGTCGTGTCC<br>R: CCTTGGAACTGCATAGTCAGG
CYP1A1	*R.norvegicus*	F: TTCAGTTCAGTCCTTCCTCACA<br>R: GAAGGCTGGGAATCCATACA
NQO1	*R.norvegicus*	F: AGCGCTTGACACTACGATCC<br>R: CGTGGGCCAATACAATCAG
LDHA	*R.norvegicus*	F: ATGAGCTTGCCCTTGTTGAT<br>R: GCGGTGATAATGACCAGCTT
SRD5A1	*R.norvegicus*	F: CGTCCTGCTGGCTATGTTTC<br>R: GGTCACCCAGTCTTCAGCAT
ß-Actin (ACTB)	*R.norvegicus*	F: AGCCATGTACGTAGCCATCC<br>R: CTCTCAGCTGTGGTGGTGAA
S14 (RPS14)	*R.norvegicus*	F: CAAGGGGAAGGAAAAGAAGG<br>R: GAGGACTCATCTCGGTCAGC

Table 4: Primers used for ChIP

GENE	SPECIE	SEQUENCE (5'-->3')	Tm (oC)	AMPLICON SIZE (bp)
AHR	*Homo sapiens*	GGGGGTGCTCCTGCTATT<br>CTCCCGGGACTTCCTAAATC	57	214
AHR -2800 Kb TSS	*Homo sapiens*	GACAATTCTGAGGGCCTGAA<br>TGAGGGAGCTTGAGCTGAGT	57	175
AHR +2500 Kb TSS	*Homo sapiens*	AGGCTAGTGCTGGCATTTTC<br>GGCTCATTAGGCCACAAACT	57	181
ARNT	*Homo sapiens*	GGCTGGCAATATCTCCCATA<br>GGTGGATTCGTCTGAGTGGT	57	238
LDHA	*Homo sapiens*	GCTTAGCAGCAGAGGGAAAA<br>CTCAGGAAGGCTTGGATCTG	57	193
LDHA -20 Kb TSS	*Homo sapiens*	TGCCTGTAGTCCCAGCTTCT<br>GGCCAGGACAATGCTTTTTA	57	166
LDHA +12 Kb TSS	*Homo sapiens*	TATTGGCTAGGCTGGTCTCG<br>GCCTAGGGTGGTCTCAAACA	57	223
LDHA	*Rattus norvegicus*	ATGTGGAGCCACCCTTACAG<br>TAAATGGAAGCTCCGTGCTG	57	158
LDHA -15 Kb TSS	*Rattus norvegicus*	GAATCCAACCCAGGACTTCA<br>ACAAAATGGCTGGTCTCCAC	57	205
NQO1	*Rattus norvegicus*	CGACACCAAAGCTGAAGAGA<br>CTGCCTGTGTGGGTTAGTCC	57	249
NOLC1 1	*Rattus norvegicus*	ATCGTGGGGTCTGAATAGCA<br>AGGACGCATGCGAAATCTAT	57	230
NOLC1 2	*Rattus norvegicus*	CTCTACCCTGGCTGTGCTCT<br>GCAAATTTACTGGCCACCTC	57	179
DDX10	*Rattus norvegicus*	GAGCACGCCTAGTTCCTCTG<br>TCAGGAGCAGCAAGGAAGAC	57	211
OGFOD1 1	*Rattus norvegicus*	TGAAGAAAAAGGGGAAACGA<br>GAGCGCACCATTAACCTGTAA	57	186
OGFOD 1 2	*Rattus norvegicus*	AGGAAGCTGGGAGGCACT<br>TTCCCGTTCATCTTCTCACC	57	239

A

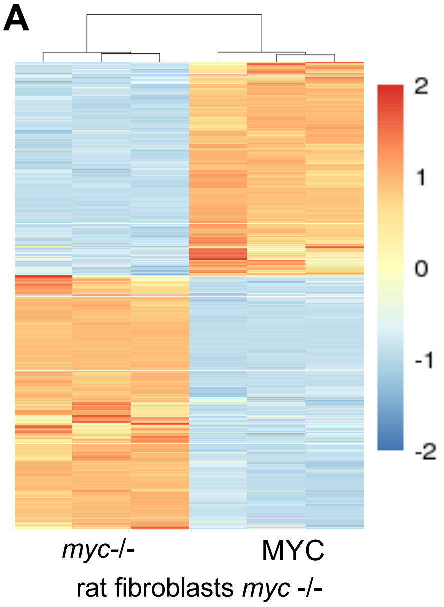

B

Genes	Up	Down
MYC vs *myc-/-*	2817	3193

Cutoff:
abs(log2FC) ≥ 0.7
p-value ≤ 0.05

D

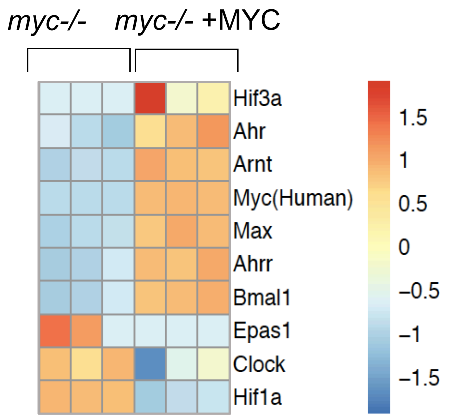

E

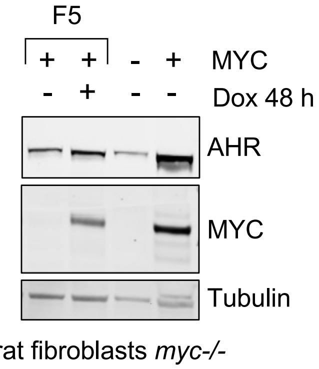

F

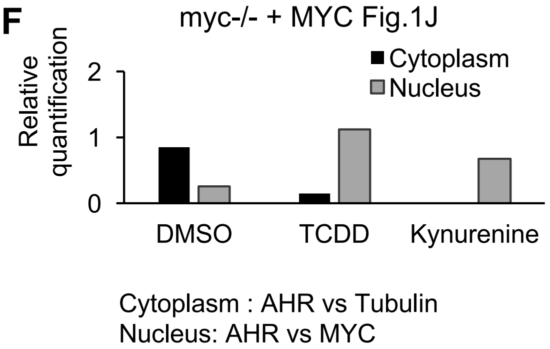

C

Pathway	Genes Overlap	q-value
MAPK Signaling Pathway	91 (54.2%)	1.02E-09
Focal Adhesion-PI3K-Akt-mTOR-signaling pathway	139 (46.2%)	9.35E-09
Focal Adhesion	91 (47.9%)	1.45E-06
Human Thyroid Stimulating Hormone (TSH) signaling pathway	41 (62.1%)	1.45E-06
Cytoplasmic Ribosomal Proteins	50 (56.8%)	2.18E-06
Adipogenesis	65 (49.6%)	1.87E-05
Spinal Cord Injury	59 (50.4%)	2.80E-05
TGF-beta Receptor Signaling	32 (58.2%)	0.000195
T-Cell antigen Receptor (TCR) Signaling Pathway	47 (51.1%)	0.000195
Nuclear Receptors Meta-Pathway	127 (40.2%)	0.000195
Oncostatin M Signaling Pathway	36 (55.4%)	0.000195
Integrated Pancreatic Cancer Pathway	74 (43.5%)	0.000697
VEGFA-VEGFR2 Signaling Pathway	97 (41.1%)	0.000697
Regulation of Actin Cytoskeleton	67 (44.4%)	0.000702
Hair Follicle Development- Induction (Part 1 of 3)	25 (59.5%)	0.000751
Apoptosis	42 (49.4%)	0.000926
Apoptotic Signaling Pathway	42 (49.4%)	0.000926
Regulation of toll-like receptor signaling pathway	63 (44.1%)	0.00122
Human Complement System	44 (47.8%)	0.00149
Extracellular vesicle-mediated signaling in recipient cells	19 (63.3%)	0.00153
B Cell Receptor Signaling Pathway	46 (46.9%)	0.00153
B Cell Receptor Signaling Pathway	46 (46.9%)	0.00153
Sudden Infant Death Syndrome (SIDS) Susceptibility Pathways	68 (42.8%)	0.00153
IL-4 Signaling Pathway	29 (53.7%)	0.00154
Photodynamic therapy-induced NF-kB survival signaling	21 (60.0%)	0.00168
Myometrial Relaxation and Contraction Pathways	66 (42.6%)	0.002
Transcription factor regulation in adipogenesis	15 (68.2%)	0.00202
RANKL-RANK (Receptor activator of NFKB (ligand)) Signaling Pathway	29 (52.7%)	0.00203
Bladder Cancer	19 (61.3%)	0.00208
Kit receptor signaling pathway	30 (50.8%)	0.00342
IL-3 Signaling Pathway	26 (53.1%)	0.00349
Toll-like Receptor Signaling Pathway	46 (45.1%)	0.00349
Senescence and Autophagy in Cancer	47 (44.8%)	0.00356
Macrophage markers	8 (88.9%)	0.00356
Chemokine signaling pathway	68 (41.2%)	0.00375
G Protein Signaling Pathways	42 (45.7%)	0.00408
IL-1 signaling pathway	28 (50.9%)	0.00415
ErbB Signaling Pathway	28 (50.9%)	0.00415
Canonical and Non-Canonical TGF-B signaling	12 (70.6%)	0.00415
Hepatitis C and Hepatocellular Carcinoma	26 (52.0%)	0.00415
Calcium Regulation in the Cardiac Cell	62 (41.6%)	0.00424
AGE-RAGE pathway	32 (48.5%)	0.00466
Brain-Derived Neurotrophic Factor (BDNF) signaling pathway	60 (41.7%)	0.00476
EGF-EGFR Signaling Pathway	66 (40.7%)	0.00527
Lung fibrosis	31 (48.4%)	0.00541
Aryl Hydrocarbon Receptor Pathway	24 (52.2%)	0.00548
Tryptophan metabolism	24 (52.2%)	0.00548
Interferon type I signaling pathways	27 (50.0%)	0.00603
Urea cycle and metabolism of amino groups	13 (65.0%)	0.00625
Hypertrophy Model	13 (65.0%)	0.00625
ESC Pluripotency Pathways	49 (42.6%)	0.00695
Prolactin Signaling Pathway	35 (46.1%)	0.00695
TYROBP Causal Network	29 (48.3%)	0.00702
Heart Development	24 (51.1%)	0.00702
White fat cell differentiation	18 (56.2%)	0.00702
IL-2 Signaling Pathway	22 (52.4%)	0.00715

Lafita-Navarro_Fig S2

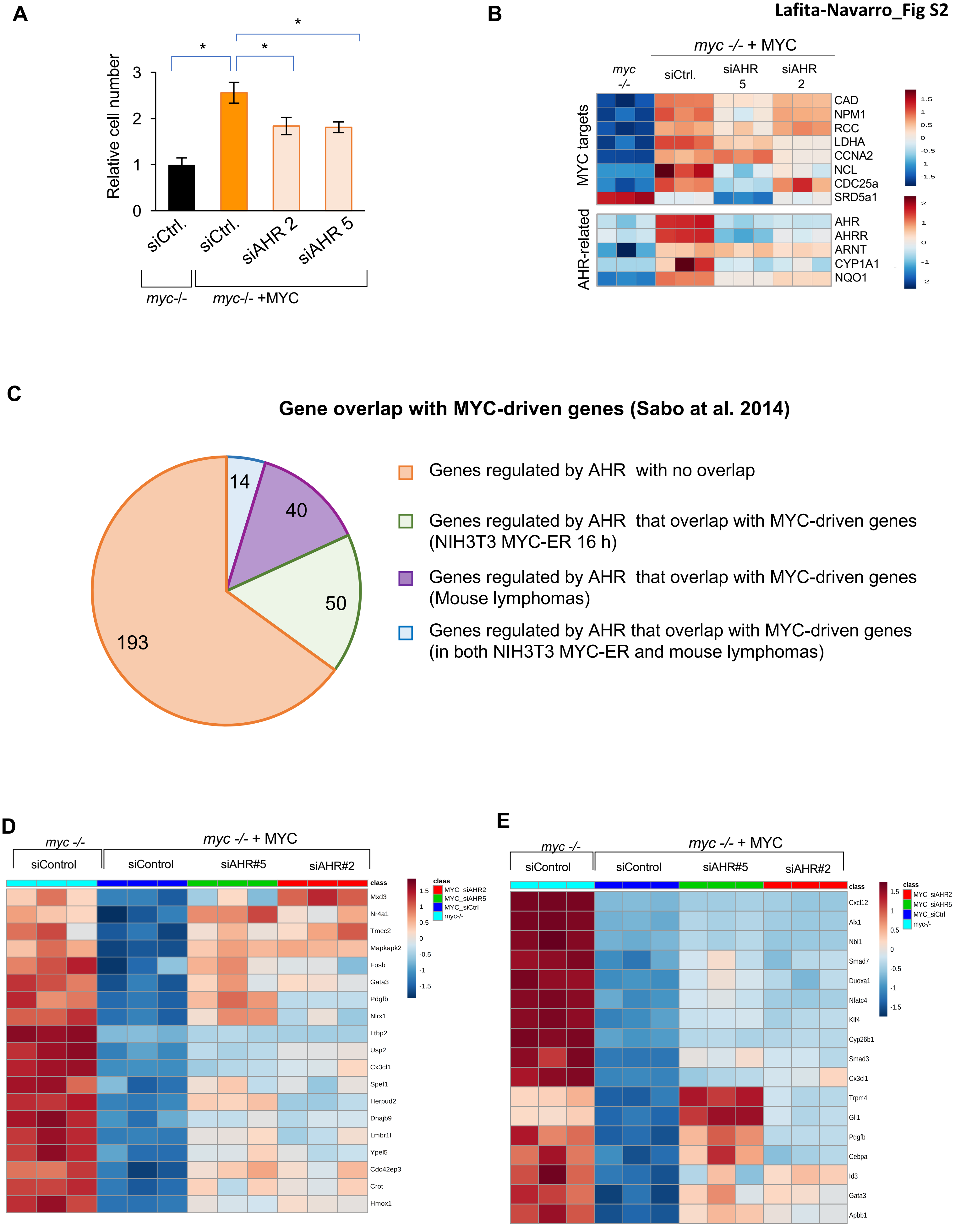

TOP 20 most significant entries with FDR q-value < 0.05
Extended graphs of Fig. 2 D-E.

A

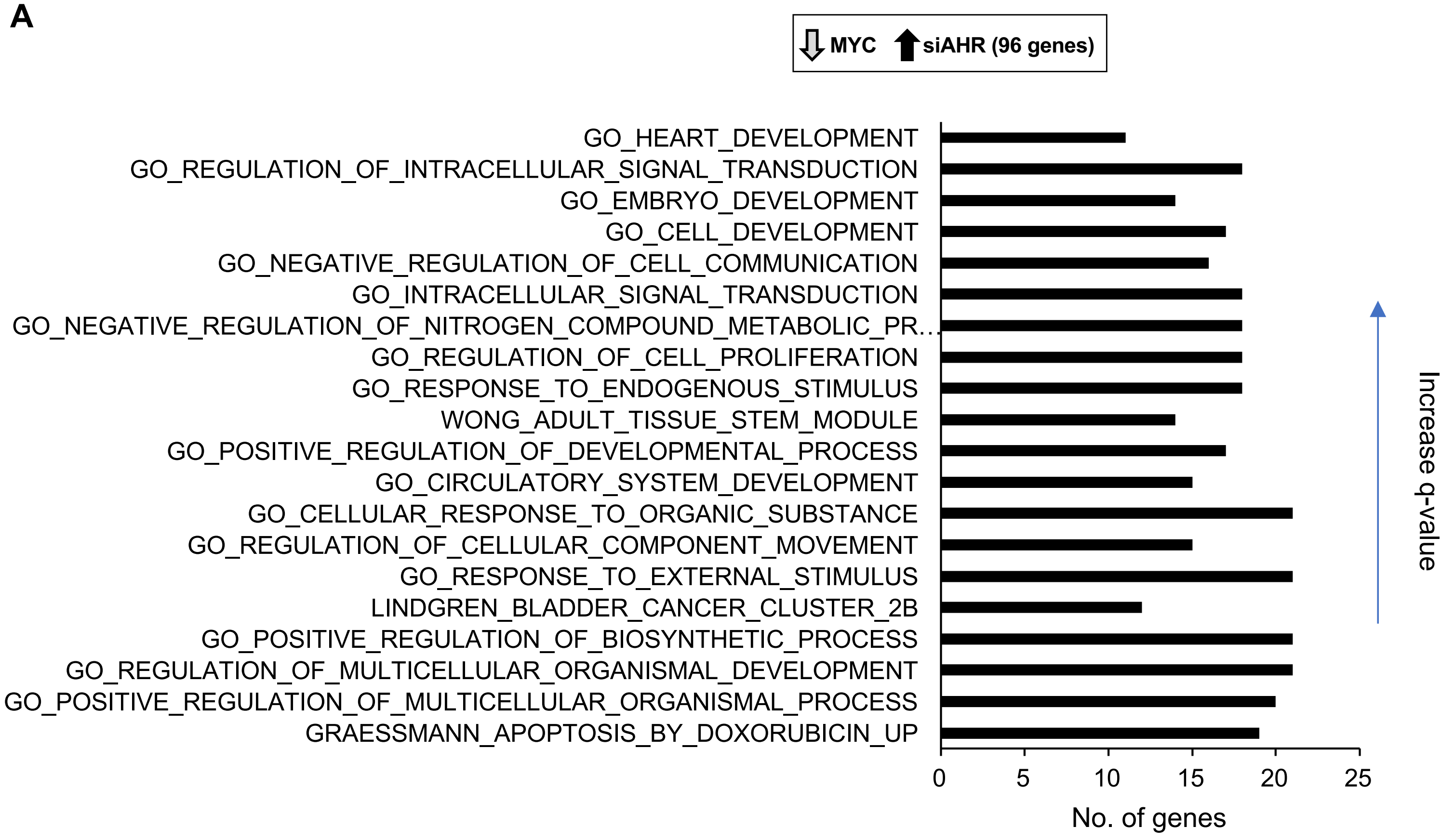

B

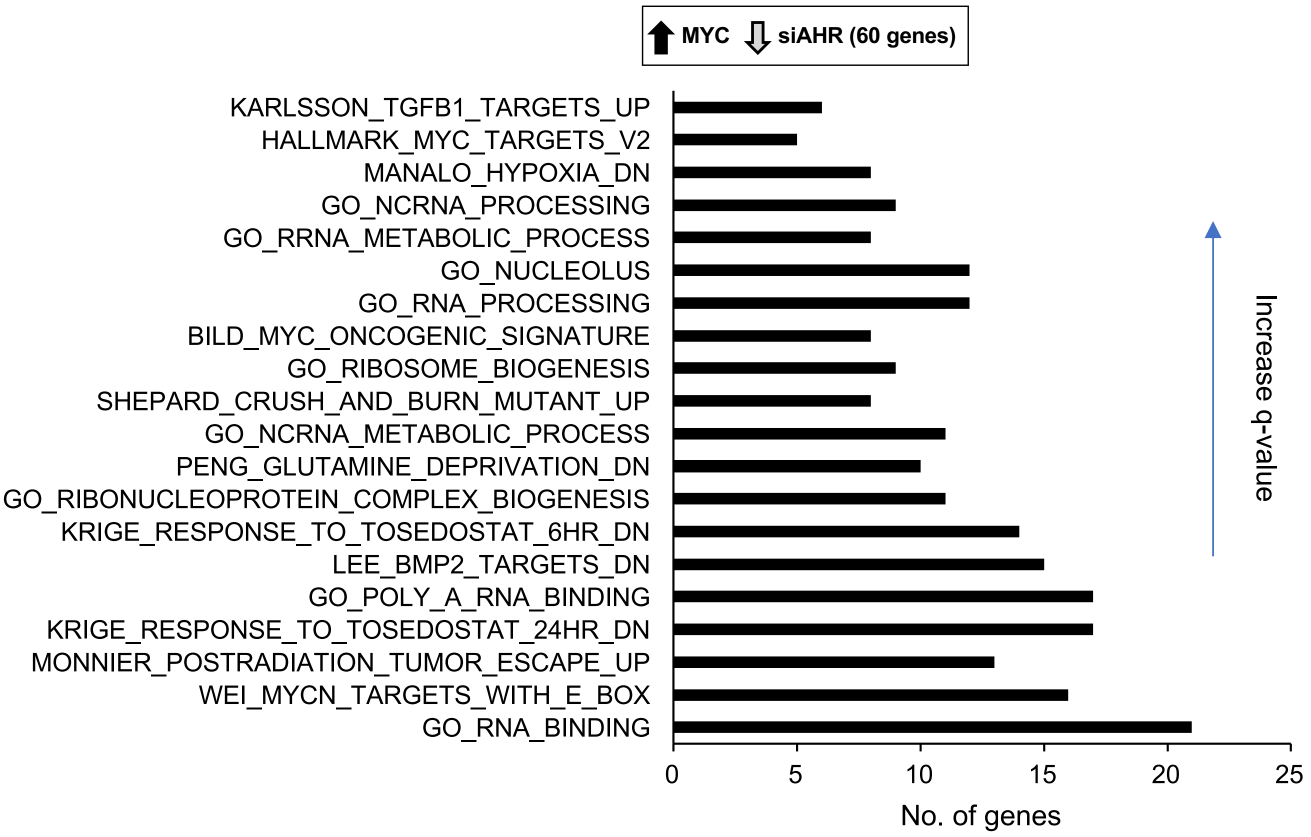

Lafita-Navarro_Fig S4

A

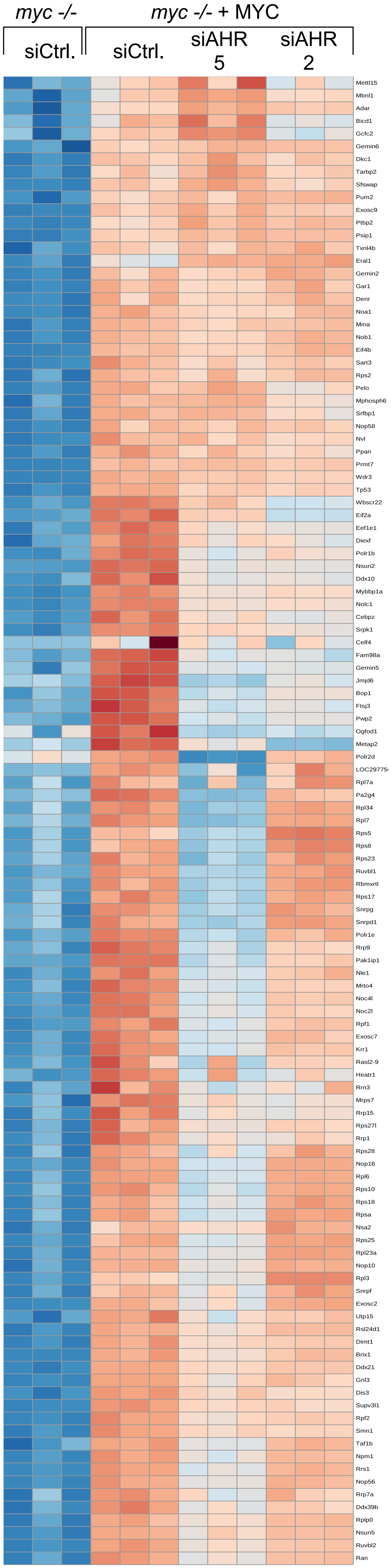

MYC-induced Ribosome processes genes

B

Gene	Function	Localization
Mybbp1a	rDNA transcription	nucleolus
Nolc1	rDNA transcription	nucleolus
Polr1b	rRNA polimerase	nucleolus
Rrp9	rRNA processing	nucleolus
Ftsj3	rRNA processing	nucleolus
Wbscr22	rRNA processing	Nucleus/nucleolus
Bop1	rRNA maturation	nucleolus
Pwp2	rRNA maturation	nucleolus
Rpf2	rRNA maturation and ribosome assembly	nucleolus
Jmjd6	RNA splicing	nucleus
Gemin5	RNA splicing	cytoplasm and nucleus
Supv3l1	RNA Helicase	mitochondria
Ddx10	RNA Helicase	nucleolus
Dis3	RNA processing	nucleus
Nsun2	Stabilization of anti-codon pairing during translation	nucleus
Fam98a	Poly(A) RNA binding	cytosol
Ogfod1	Translation regulation	nucleus
Eif2a	Translation Initiation	cytosol
Eef1e1	Translation elongation	cytosol
Metap2	Translation regulation	cytosol

C

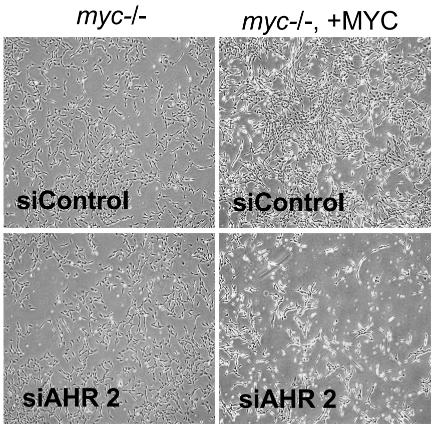

D

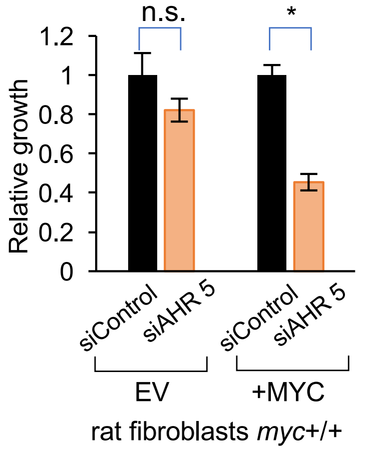

E

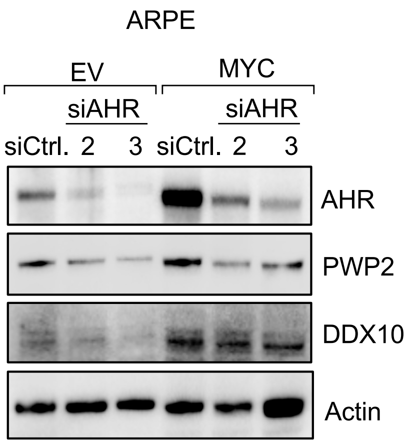

F

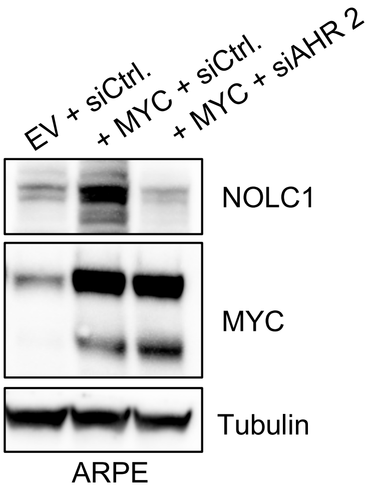

G

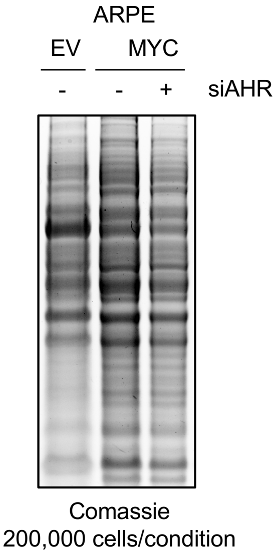

H

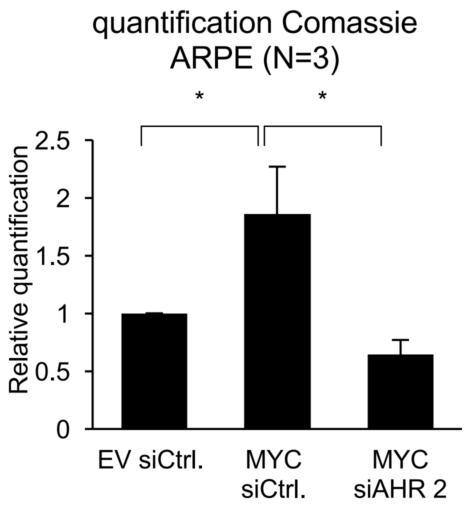

Lafita-Navarro_Fig S5

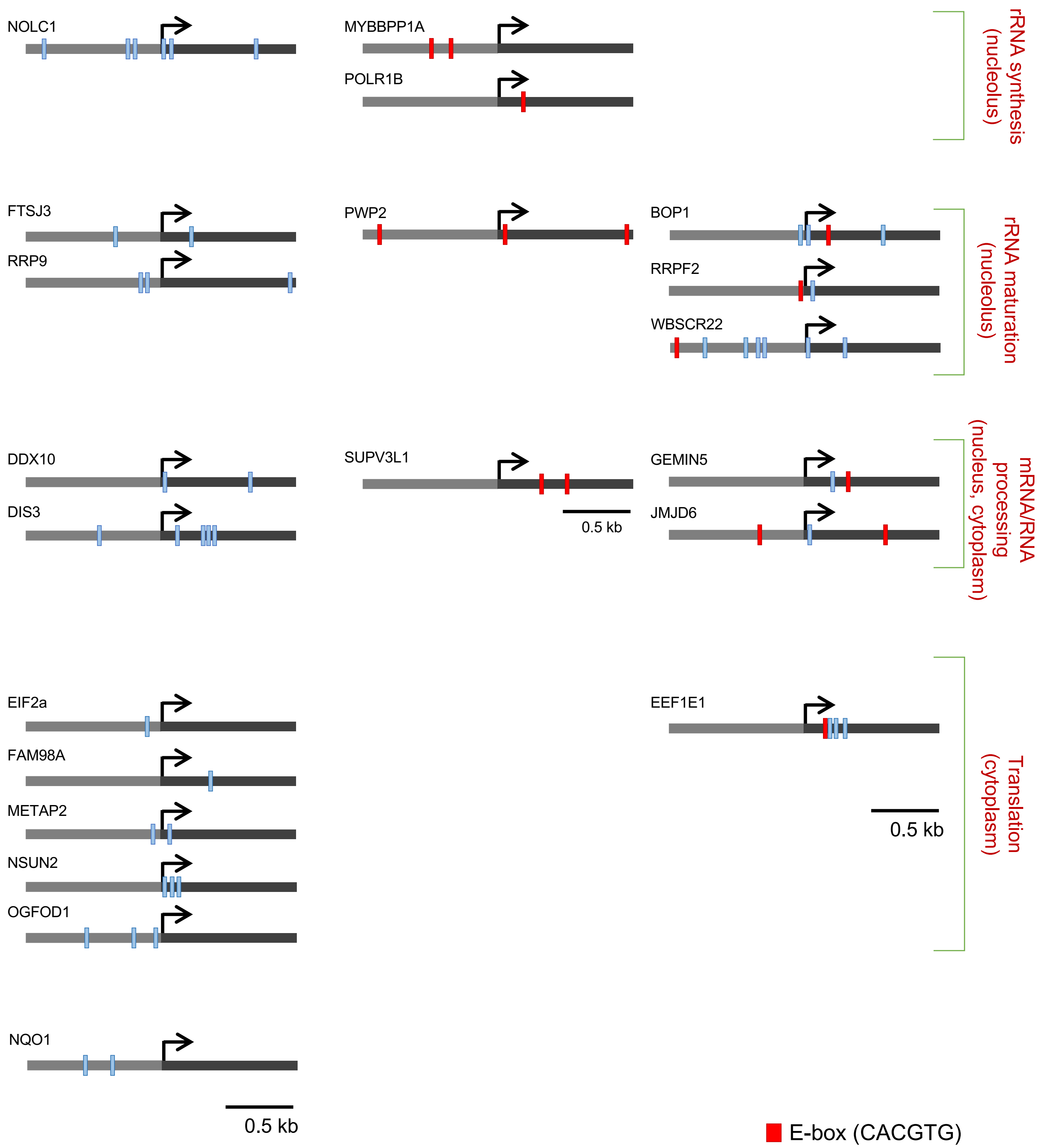

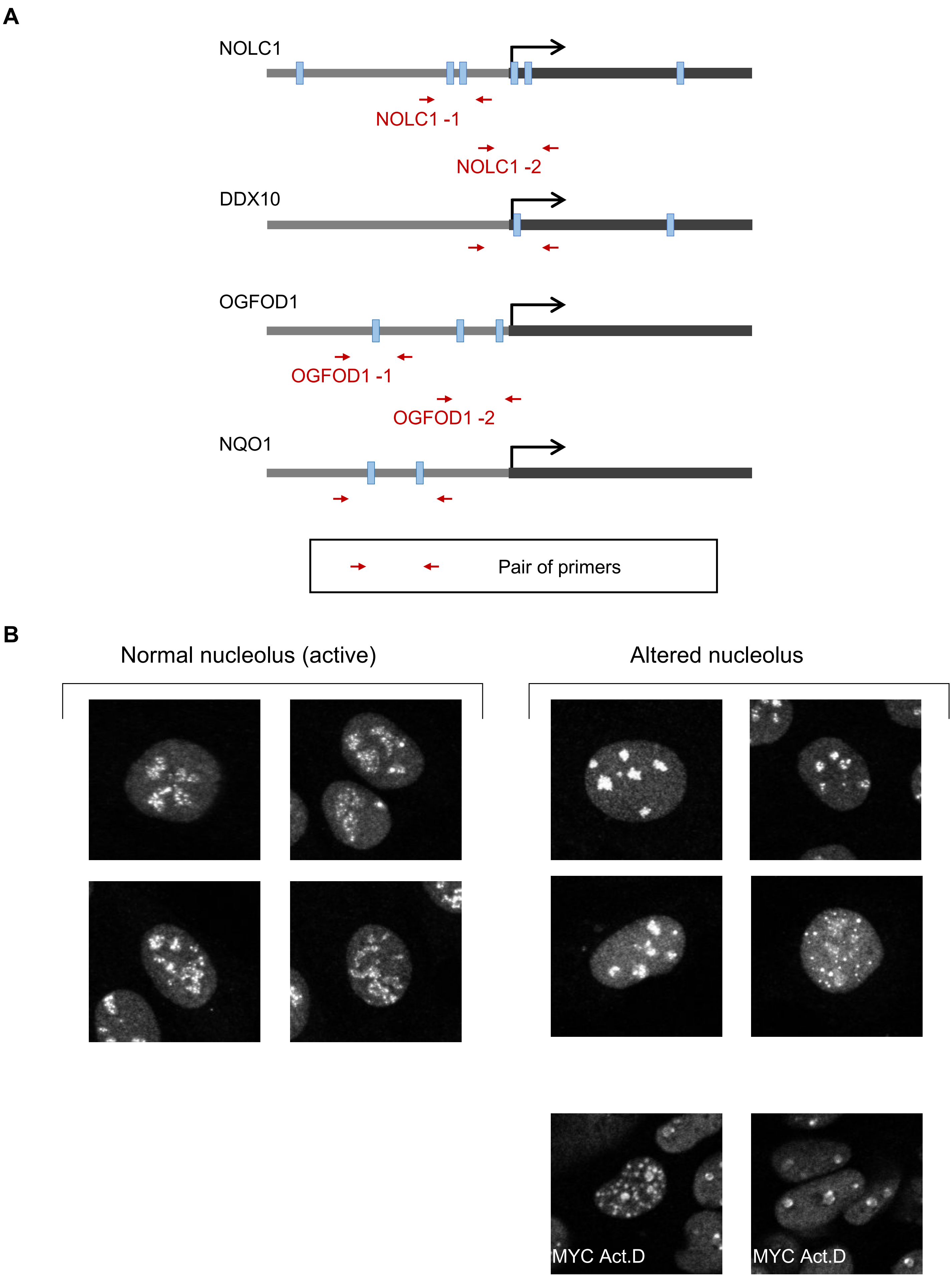
A
NOLC1
NOLC1 -1
NOLC1 -2
DDX10
OGFOD1
OGFOD1 -1
OGFOD1 -2
NQO1
Pair of primers
B
Normal nucleolus (active)
Altered nucleolus
MYC Act.D
MYC Act.D

Lafita-Navarro_Fig S7

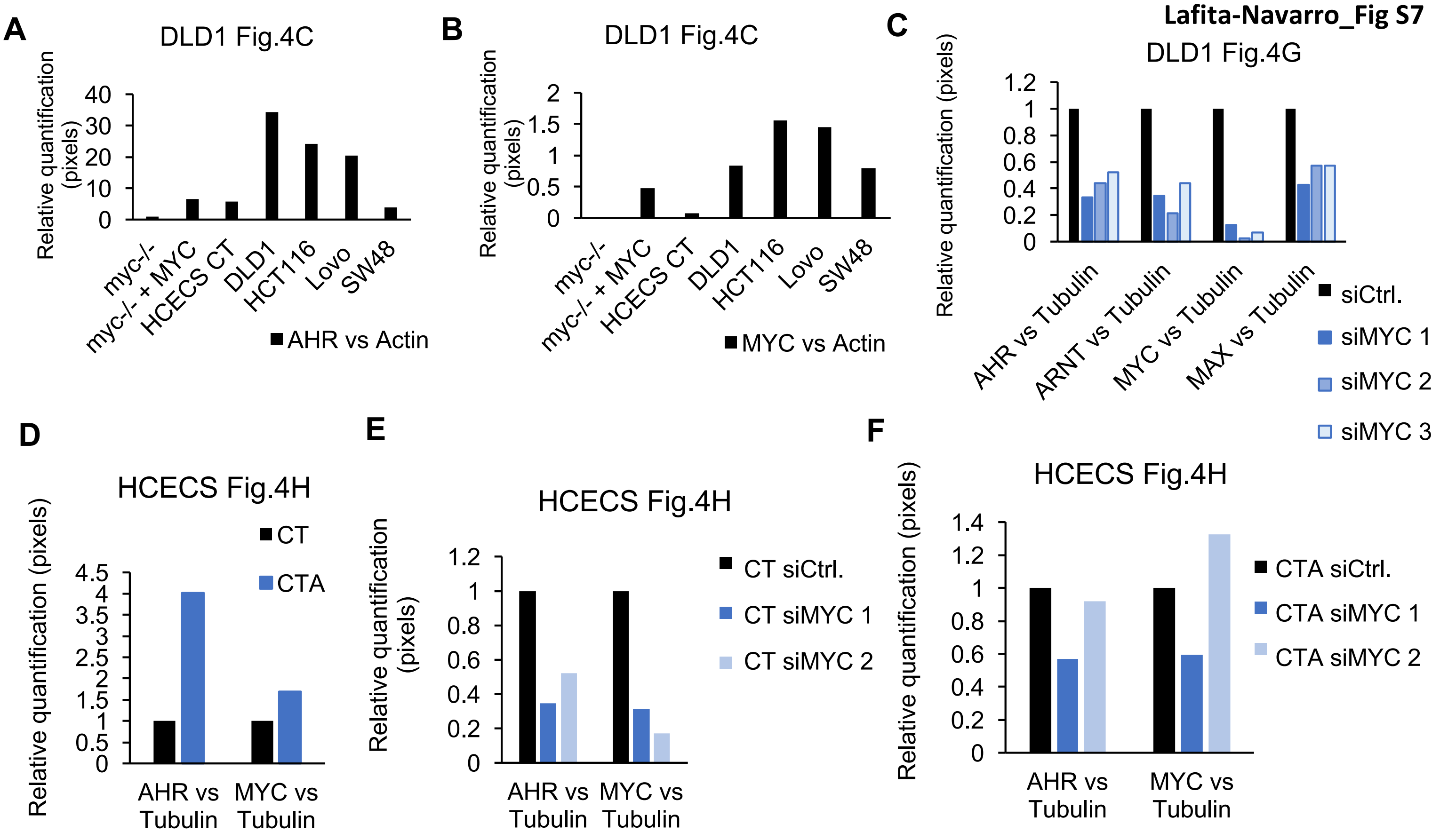

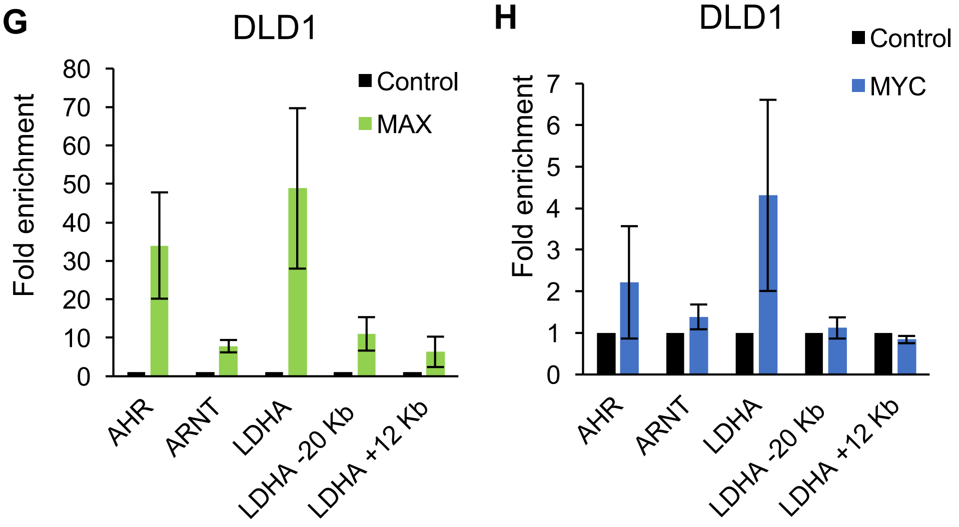

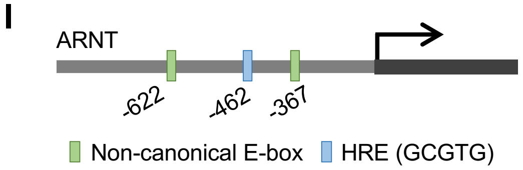

J

	Promoter region			
	AHR		ARNT	
cell line	MYC	MAX	MYC	MAX
K562	-	-	+	+
A549	+	+	+	+
H1ES	+		+	
HeLa	+	+	+	+
NB4	+	+	+	+
GM12878		+		+
HEPG		+		+
HUVEC		+		+

A

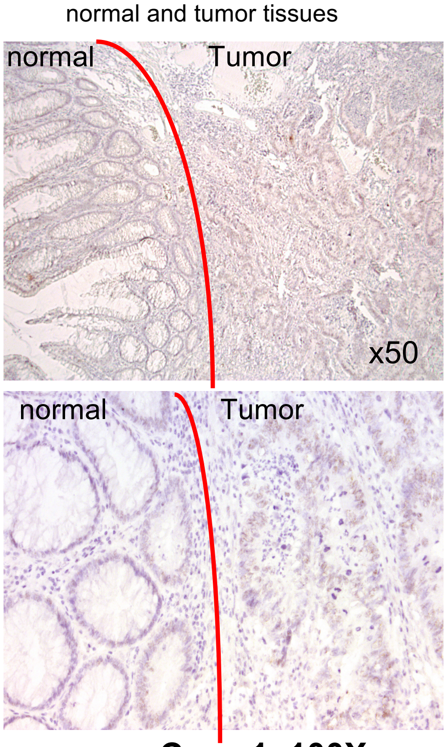

Case 1 100X

B

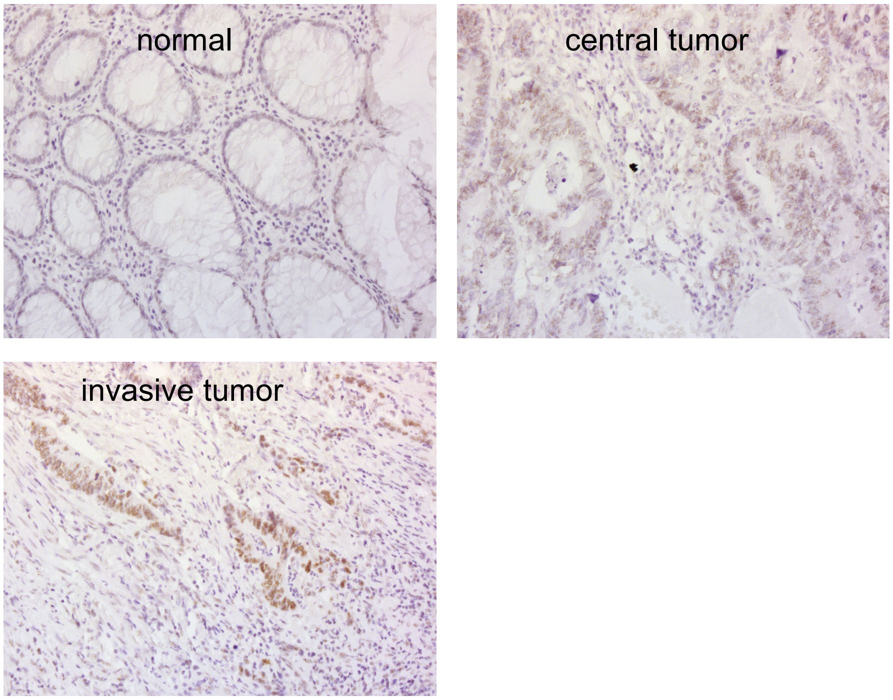

Case 1 200X

C

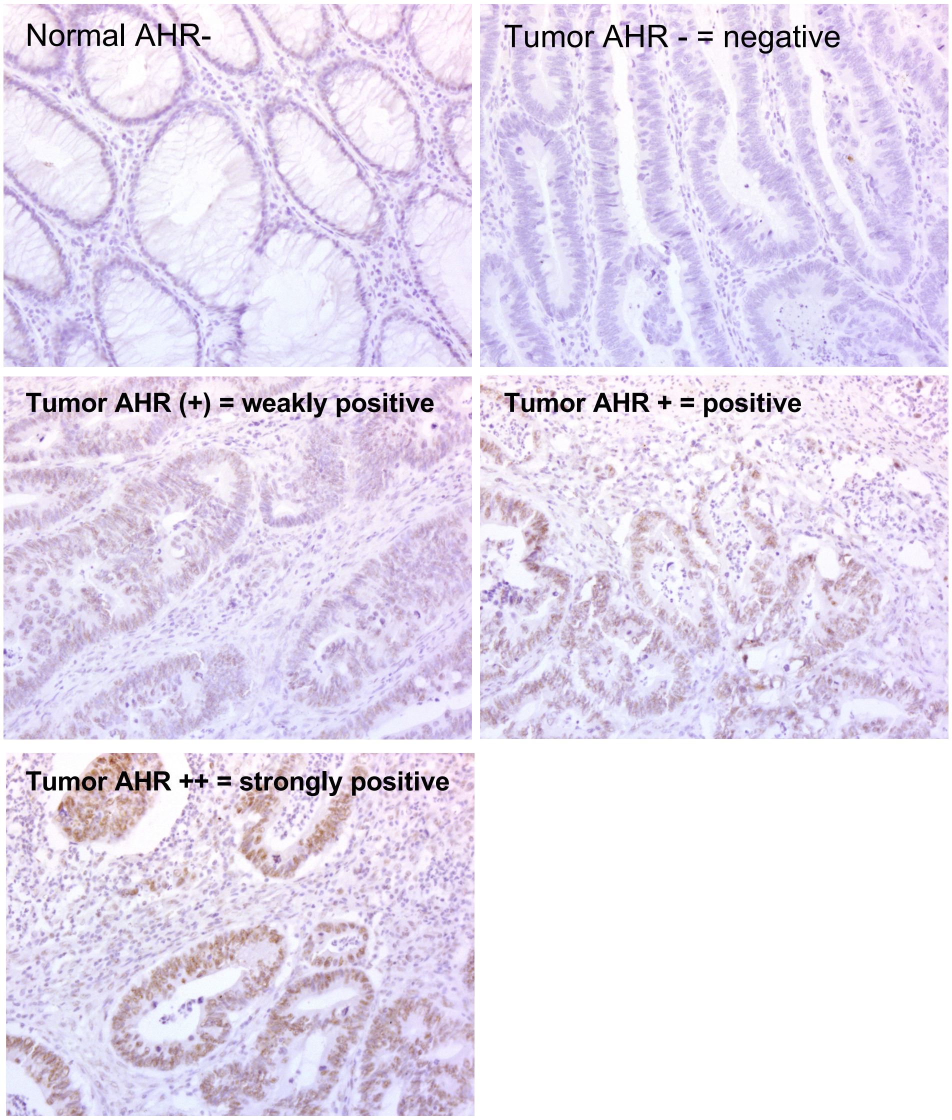

D

Case no.	AHR
1	n ++
2	n ++
3	n (+)
4	n +
6	n (+)
7	n ++
9	n +
10	-
11	-
13	n +
14	n ++
15	-
16	n ++
17	n +
18	-
20	n ++
21	n ++
22	n +

n = nuclear
++ = strongly positive
+ = positive
(+) = weakly positive

Lafita-Navarro_Fig S9

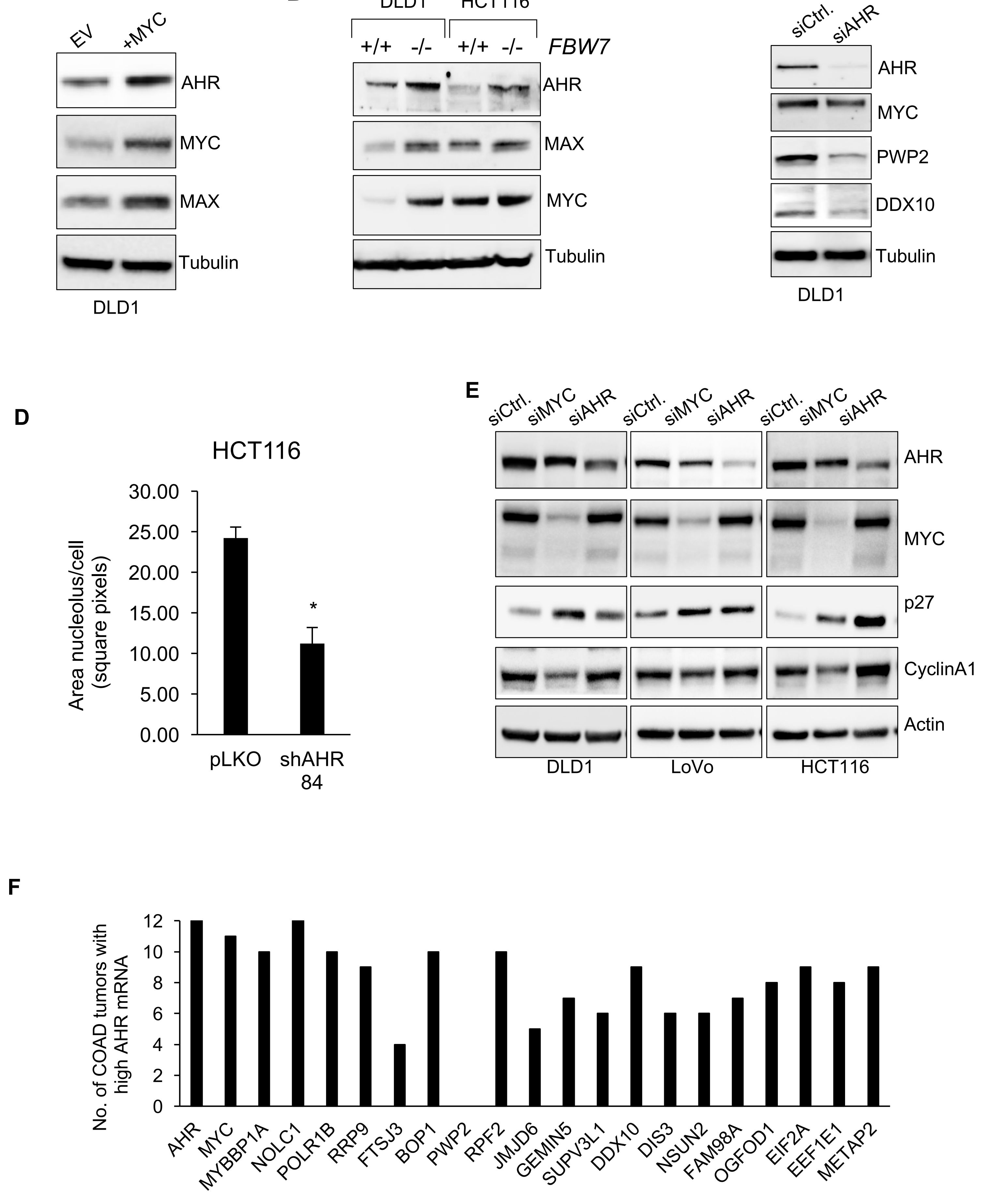

Supplement: Supplemental Material [file supp_gad.313007.118_Supplementary_figures_and_legends.pdf]
